# Decreased Memory and Learning Ability Mediated by Bmal1/M1 Macrophages/Angptl2/Inflammatory Cytokine Pathway in Mice Exposed to Long-Term Blue Light Irradiation

**DOI:** 10.3390/cimb46050295

**Published:** 2024-05-18

**Authors:** Keiichi Hiramoto, Sayaka Kubo, Keiko Tsuji, Daijiro Sugiyama, Hideo Hamano

**Affiliations:** 1 Department of Pharmaceutical Sciences, Suzuka University of Medical Science, Suzuka 513-8670, Mie, Japan; 2 Research Department, Daiichi Sankyo Healthcare Co., Ltd., Chuo-ku 140-8170, Tokyo, Japan; kubo.sayaka.hr@daiichisankyo-hc.co.jp (S.K.); tsuji.keiko.nj@daiichisankyo-hc.co.jp (K.T.); sugiyama.daijiro.gz@daiichisankyo-hc.co.jp (D.S.); hamano.hideo.gg@daiichisankyo-hc.co.jp (H.H.)

**Keywords:** blue light, brain and muscle arnt-like 1, inducible nitric oxide synthase, arginase-1, angiopoietin like protein 2, interleukin-6

## Abstract

Humans are persistently exposed to massive amounts of blue light via sunlight, computers, smartphones, and similar devices. Although the positive and negative effects of blue light on living organisms have been reported, its impact on learning and memory remains unknown. Herein, we examined the effects of widespread blue light exposure on the learning and memory abilities of blue light-exposed mice. Ten-week-old male ICR mice were divided into five groups (five mice/group) and irradiated with blue light from a light-emitting diode daily for 6 months. After 6 months of blue light irradiation, mice exhibited a decline in memory and learning abilities, assessed using the Morris water maze and step-through passive avoidance paradigms. Blue light-irradiated mice exhibited a decreased expression of the clock gene brain and muscle arnt-like 1 (Bmal1). The number of microglia and levels of M1 macrophage CC-chemokine receptor 7 and inducible nitric oxide synthase were increased, accompanied by a decrease in M2 macrophage arginase-1 levels. Levels of angiopoietin-like protein 2 and inflammatory cytokines interleukin-6, tumor necrosis factor-α, and interleukin-1β were elevated. Our findings suggest that long-term blue light exposure could reduce Bmal1 expression, activate the M1 macrophage/Angptl2/inflammatory cytokine pathway, induce neurodegeneration, and lead to a decline in memory.

## 1. Introduction

In recent years, there has been a marked increase in human exposure to blue light (380–495 nm visible light) from light-emitting diodes (LED), fluorescent or incandescent lights, computer and smartphone screens, and other devices [1,2].

Blue light has been shown to induce several effects on humans. Specifically, prolonged exposure to blue light can induce dry eyes [3,4], blurred vision [4,5], eye fatigue [6], retinal damage, and age-related macular degeneration [7].

In addition, blue light is associated with circadian rhythms, which are involved in melatonin secretion at night. Melatonin level is strongly correlated with sleep, with high melatonin levels inducing sleep. Blue light acts on the brain and induces wakefulness by suppressing melatonin secretion [8]. In fact, blue light suppresses melatonin secretion even under relatively dim lighting (50–100 lx) [9]. Therefore, using a smartphone or computer at night is likely to cause sleep disorders. Moreover, circadian rhythm disturbances caused by blue light exposure increase the risk of cancer [10], obesity [11], diabetes [12], hypertension [13], and depression [14,15]. Blue light exposure can also accelerate aging [16] and damage brain cells [17]. However, despite these blue light-mediated negative effects on living organisms, it is also important for regulating circadian rhythms and sleep and for maintaining overall health. Blue light exposure has also been associated with improved sleep quality, brain function, and increased brain volume [18]. Therefore, the effects of blue light on humans are yet to be comprehensively elucidated.

In a previous study, we found that irradiation of eyes with ultraviolet A waves (UVA) could cause a decline in memory and learning abilities in mice [19]. Long-term UVA ocular irradiation decreased acetylcholine levels; increased γ-secretase, β-amyloid, and advanced glycation end products in the brain; and decreased glucose uptake into the brain [19]. Moreover, mice subjected to long-term UVA ocular irradiation exhibited hippocampal degeneration [20]. However, the effect of blue light, which has a longer wavelength than UVA and can reach the retina and affect the brain, on memory and learning ability remains elusive.

Accordingly, the purpose of this study was to determine the effects of daily blue light exposure over a 6-month period on the memory and learning abilities of mice and elucidate how blue light affects the brain.

## 2. Materials and Methods

### 2.1. Animal Experiments

**Figure 1:**
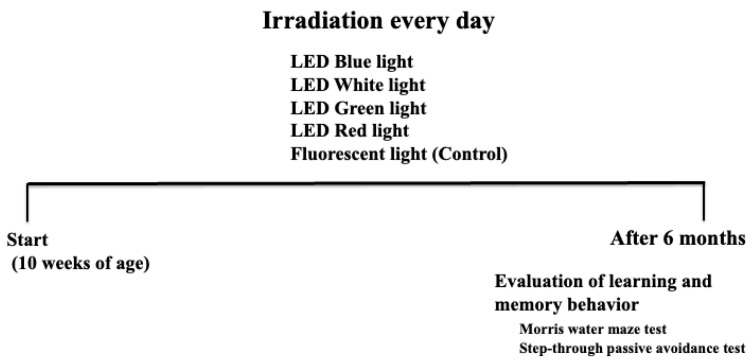
Schematic diagram of the experimental method.

**Figure 2:**
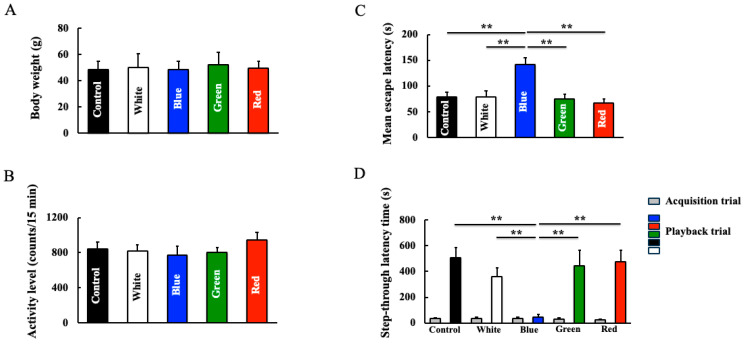
Effect of long-term blue light irradiation on body weight (**A**), motor activity (**B**) memory and learning ability (**C**,**D**) of mice. The Morris water maze test (**C**) and step-through passive avoidance test (**D**) were used to the assess the memory and learning ability of mice. Data values are expressed as the mean ± standard deviation (SD) derived from five specimens. ** *p* < 0.01.

We used 10-week-old male, specific-pathogen-free (SPF) Institute of Cancer Research mice (SLC, Hamamatsu, Shizuoka, Japan) as experimental animals. The groups of mice were housed in cages in an air-conditioned room maintained at 23 ± 1 °C with a 12 h light/12 h dark cycle under SPF and stress-free conditions. Mice were divided into five groups, comprising five mice each: control, white light irradiation, blue light irradiation, green light irradiation, and red light irradiation. The following light sources were used: fluorescent lamp, LED blue light (wavelength: 380–500 nm, peak emission: 479 nm, 40 kJ/m^2^, ISLM-150 × 150-BB; CCS, Kyoto, Japan), LED green light (wavelength: 500–560 nm, peak emission: 538 nm, 40 kJ/m^2^, ISLM-150X150-GG; CCS, Kyoto, Japan), LED red light (wavelength: 600–700 nm, peak emission: 629 nm, 40 kJ/m^2^, ISLM-150X150-RR; CCS, Kyoto, Japan), and LED white light (12 kJ/m^2^, ISLM-150X150-HWW; CCS, Kyoto, Japan). The energy content of each LED light was measured using a light analyzer (LA-105; Nippon Medical & Chemical Instruments, Osaka, Japan). The control group was irradiated using a fluorescent lamp typically used for breeding purposes. For a specific treatment group, the entire body of each mouse was exposed to the corresponding LED light daily (10 min/day) for 6 months [18] (Figure 1). Irradiation was performed at a constant time, 10:00 a.m., during the test period. There was no difference between the LED green light and red light when compared with the fluorescent light. This could explain why blue light irradiation exerts a stronger effect on learning and memory ability than white light, yellow light, or red light, as mice are most sensitive to the range of UV to blue light to green light [21]. Therefore, the examinations from Figure 2 onward were conducted using three groups: control, white light, and blue light. In addition, this study was retested once, and the results were equivalent. This study was approved by the Suzuka University of Medical Science Animal Experiment Ethics Committee on 25 September 2014, and was performed in strict accordance with the recommendations of the Guide for the Care and Use of Laboratory Animals of Suzuka University of Medical Science (Approval number: 34). All surgeries in mice were performed under pentobarbital anesthesia, and efforts were made to minimize animal suffering.

### 2.2. Spontaneous Locomotor Activity

Locomotor activity was measured using a locomotor activity measurement device (Melquest, Toyama, Japan), and the amount of locomotor activity was measured and quantified using an infrared sensor attached to the bottom of the cage. Using the normal rearing cage as the measurement cage, one mouse from each treatment group was placed in a new environment (no training), and their locomotor activities were measured for 20 min. In the current study, the locomotor activity-measuring device used a sensor to measure the number of times the mouse moved a certain distance, allowing only individual measurements at a time.

### 2.3. Morris Water Maze Test

This test was adapted from a previously published method by Morris [22]. The spatial learning ability of the mice was first evaluated for 5 consecutive days through hidden platform training; there were four trials per day. On Day 6, the platform was removed, and the mice were individually tested for spatial memory by conducting single 60 s probe trials. Swim paths were recorded, and the latency to reach the platform during water maze training, number of crossings over the former platform location (target area) during the probe trial, and time spent in the target quadrant during the probe trial were analyzed.

### 2.4. Step-Through Passive Avoidance Test

This test was performed to evaluate nonspatial long-term memory using a previously described method [23]. The experimental apparatus (Bio Research Center, Nagoya, Aichi, Japan) consisted of two compartments: a light compartment and a dark compartment, separated by a grid door. A stainless-steel shock grid floor was placed in the dark compartment. During the acquisition trial, mice were placed in the light compartment. After 60 s, the grid door between the compartments was opened. The step-through latency for the mice to enter the dark compartment was measured, and the door was closed. Immediately after the mice entered the dark compartment, an inescapable foot shock (0.5 mA for 3 s) was applied. The retention test was performed 24 h after the training trial in a similar manner without the electric shock, and the step-through latency to enter the dark compartment was recorded. The maximum cutoff time for step-through latency was 600 s. The step-through passive avoidance test was performed after the water maze test was completed, with an interval of 10 days to eliminate the influence of the water maze test.

### 2.5. Measurement of Inducible Nitric Oxide Synthase (iNOS), Arginase-1 (Arg-1), Angiopoietin like Protein 2 (ANGPTL2), Interleukin (IL)-6, Tumor Necrosis Factor (TNF)-α, and IL-1-β Levels in Hippocampus

Hippocampal tissue samples were collected on the final day of the experiment. The hippocampus was isolated and homogenized in a lysis buffer (Kurabo, Osaka, Japan). The tissue extracts were centrifuged (Tomy MX-201, TOMY DIGITAL BIOLOGY Co., Ltd., Nerima-ku, Tokyo, Japan) at 10,000 rpm, and supernatants were collected to perform the assay. Commercial enzyme-linked immunosorbent assay kits were used, according to the manufacturers’ instructions, to measure the following: iNOS and Arg-1 (CUSABIO, Houston, TX, USA), ANGPTL2 (biorbyt, Cambridge, UK), IL-6 (RayBiotech Life, Peachtree Corners, GA, USA), TNF-α (Enzo Life Sciences, Farmingdale, NY, USA), and IL-1β (Abcam, Cambridge, MA, USA). Optical density was measured using a microplate reader (Molecular Devices; Sunnyvale, CA, USA).

### 2.6. Western Blotting Analysis of the Hippocampus Tissue Specimens

Hippocampal tissue samples were homogenized in a lysis buffer (Kurabo, Osaka, Japan). The homogenates were centrifuged, and the supernatants were obtained. Western blotting was performed as previously described [24]. The membranes were incubated at room temperature for 1 h with primary antibodies against brain and muscle arnt-like 1 (Bmal1) (1:1000; Cell Signaling Technology, Danvers, MA, USA), Cryptochrome 1 (Cry1) (1:1000; Proteintech Group, Rosemont, IL, USA), Cry2 (1:1000; Proteintech Group, Rosemont, IL, USA), ionized calcium-binding adapter protein 1 (Iba1; marker of microglia (1:1000; Wako, Osaka, Japan), CC-Chemokine receptor 7 (CCR7) (1:1000; Abcam; Cambridge, MA, USA), and β-actin (1:5000; Sigma-Aldrich, St. Louis, MO, USA) as loading controls. The membranes were washed and incubated with horseradish peroxidase-conjugated secondary antibody (Novex, Frederick, MD, USA). Immune complexes were detected using ImmunoStar Zeta reagent (Wako, Osaka, Japan), and images were captured using Multi Gauge Software ver. 3.0 (Fujifilm, Greenwood, SC, USA).

### 2.7. Statistical Analysis

Data obtained from the experiments are presented as mean ± standard deviation (SD). The data were analyzed using Microsoft Excel for Mac ver. 16.78 (Microsoft Corp., Redmond, WA, USA); one-way analysis of variance followed by Tukey’s post hoc test was performed using SPSS version 20 (SPSS, Chicago, IL, USA). Differences with *p*-values * <0.05, ** <0.01 were considered significant.

## 3. Results

### 3.1. Behavioral Effects of Long-Term Blue Light Irradiation in Mice

The body weights (Figure 2A) and locomotor activity (Figure 2B) of mice at the end of the experiment did not differ between the control and LED light groups. In the Morris water maze assessment (spatial memory and learning), blue light-irradiated mice showed a decline in learning and memory (Figure 2C). No differences were observed between the other irradiation groups and the control group. Furthermore, in the step-through latency test (experiential learning and memory), blue light-irradiated mice exhibited a decline in learning ability and memory; meanwhile, no differences were observed between the other LED groups and the control group (Figure 2D).

### 3.2. Effect of Long-Term Blue Light Irradiation on the Expression of Bmal1, Cry1, and Cry2 in the Hippocampus

**Figure 3:**
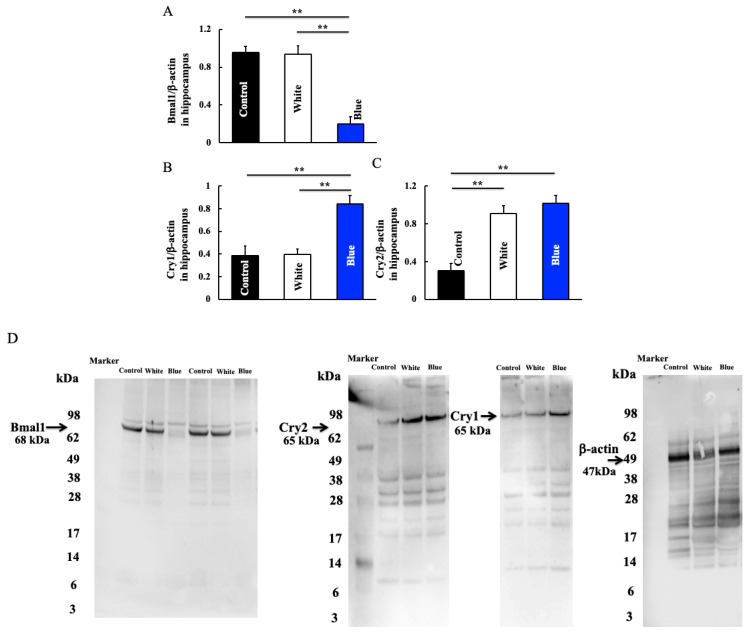
Effect of long-term blue light irradiation on the expression of Bmal1 (**A**), Cry1. (**B**), and Cry2 (**C**) in the hippocampus of mice. Western blot diagram of Bmal1, Cry1, and Cry2 with molecular weight markers (**D**). Data values are expressed as the mean ± standard deviation (SD) derived from five specimens. ** *p* < 0.01.

We detected the hippocampal expression of clock genes that are related to blue light. Blue light irradiation for 6 months reduced the hippocampal expression of Bmal1 (Figure 3A,D). Conversely, expression levels of Cry1 (Figure 3B,D) and Cry2 (Figure 3C,D), which are involved in the expression of Bmal1, increased after blue light irradiation.

### 3.3. Effect of Long-Term Blue Light Irradiation on Iba1, CCR7, iNOS, and Arg-1 Levels in the Hippocampus

**Figure 4:**
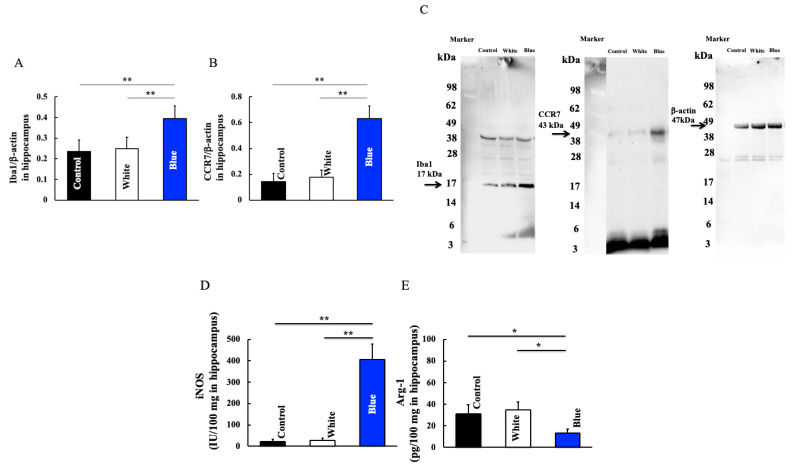
Effect of long-term blue light irradiation on the expression of Iba1 (**A**), CCR7 (**B**), iNOS (**D**), and Arg-1 (**E**) in the hippocampus of mice. Western blot diagram of Iba1 and CCR7 with molecular weight markers (**C**). Data values are expressed as mean ± standard deviation (SD) derived from five specimens. * p < 0.05; ** *p* < 0.01.

Next, we examined the expression of genes correlated with immune cell populations that are involved in memory and learning. Blue light irradiation for 6 months increased the expression of Iba1, which are hippocampal macrophages (Figure 4A,C). Furthermore, macrophages are divided into two types: M1 and M2. Blue light irradiation increased the levels of CCR7 and iNOS, which are expressed by M1 macrophages (Figure 4B–D). In contrast, the level of Arg-1, a gene expressed by M2 macrophages, was decreased by blue light irradiation (Figure 4E). This data could indicate an altered M1/M2 ratio in the hippocampus after blue light exposure.

### 3.4. Effect of Long-Term Blue Light Irradiation on Angptl2, IL-6, TNF-α, and IL-1β Levels in the Hippocampus

**Figure 5:**
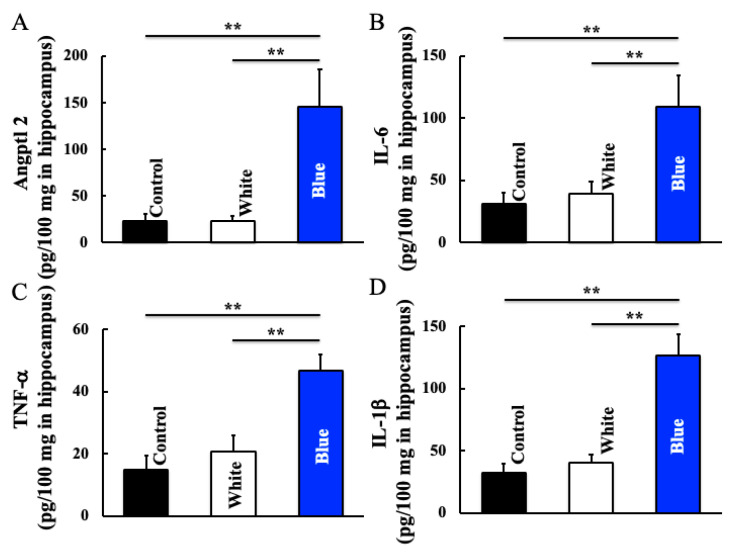
Effect of long-term blue light irradiation on the expression of Angptl2 (**A**), IL-6 (**B**), TNF-α (**C**), and IL-1β (**D**) in the hippocampus of the mice specimens. The values are expressed as means ± SD derived from five specimens. ** *p* < 0.01.

Furthermore, we detected the expression levels of genes induced by macrophages. Blue light irradiation increased hippocampal levels of Angptl2 (Figure 5A), IL-6 (Figure 5B), TNF-α (Figure 5C), and IL-1β (Figure 5D).

## 4. Discussion

In the current study, the memory and learning abilities of mice declined after blue light irradiation for 6 months. Expression levels of clock genes Bmal1 and Cry1,2 showed a tendency to decrease and increase, respectively, in blue light-irradiated mice. Additionally, the number of microglia and M1 type macrophages (CCR7) were increased. Furthermore, levels of Angptl2, IL-6, TNF-α, and IL-1β showed a tendency to increase following blue light irradiation.

Blue light irradiation induces a decrease in Bmal1 expression and an increase in Cry1 expression [25]. Consistently, we found that blue light irradiation could induce a decrease in Bmal1 expression and an increase in Cry1/2 expression (Figure 3). Furthermore, an increase in M1 type macrophage was also observed (Figure 4). Although this study did not directly investigate the signal transduction between Bmal1 and M1 type macrophages, Bmal1 reportedly regulates macrophages [26] and mitochondrial metabolism and produces mitochondrial reactive oxygen species (mROS) under metabolic stress [27,28]. mROS stabilizes hypoxia-inducible factor-1a [29], which is required for Arg-1 expression [26]. M1 macrophages exhibit deleterious functions, whereas M2 macrophages act as anti-inflammatory agents [30,31]. Arg-1 and iNOS are markers of M2 and M1 macrophages, respectively; therefore, an increase in Arg-1 levels indicates an increase in the number of M2 macrophages. Herein, blue light irradiation induced a reduction in Bmal1 levels. Therefore, we speculate that levels of Arg-1 decreased and those of iNOS increased, leading to a shift to M1 macrophages. M1 macrophages secrete the inflammatory cytokines IL-1β, IL-6, and TNF-α.

Furthermore, Bmal1 controls NF-E2-related factor-2 (Nrf2) mRNA expression and activity through an E-box that binds directly to its promoter [32]. Nrf2 controls inflammation by suppressing ROS and directly suppressing IL-1β and IL-6. Therefore, when Bmal1 expression decreases, ROS accumulation in macrophages increases, and Nrf2 activity decreases; this may enhance the production of the pro-inflammatory cytokine IL-1β [33].

On the other hand, Angptl2 is induced during tissue damage repair and is crucial for maintaining homeostasis in the body [34,35]. Furthermore, Angptl2 promotes the degradation of inhibitor of nuclear factor-κB (I-κB), a nuclear factor-κB (NF-κB) suppressor gene, through α5β1 integrin, causing nuclear translocation of NF-κB and activating the expression of inflammation-related genes [36,37]. As a result, Angptl2 promotes the activation of p38 mitogen-activated kinase (MAPK) and provokes the expression of matrix metalloproteinases (MMPs) [36,38]. In the current study, blue light irradiation increased Angptl2 levels in the hippocampus (Figure 5). Furthermore, abundant Angptl2 expression has been detected in macrophages [39]. High expression levels of Angptl2 in macrophages accumulated within lesions induce a hyperreactive state of tissue repair mechanisms, such as activation of MMPs and chronic inflammation [40]. In the current study, the increased hippocampal levels of Angptl2 suggest that Angptl2 enhanced inflammatory cytokines in hippocampal glial cells.

Macrophages are typically divided into type 1 and type 2. M1 macrophages secrete inflammatory cytokines such as TNF-α and IL-6, whereas M2 macrophages secrete anti-inflammatory cytokines such as IL-4 and TGF-β [41]. M1 macrophages activated in this manner increase the inflammatory cytokine TNF-α, both directly and indirectly. TNF-α induces M1 microglia and further enhances the secretion of inflammatory cytokines, including iNOS and CCR7. M1 macrophages and TNF-α form a loop that leads to a vicious cycle [31]. Furthermore, an increase in M1 type macrophages was also observed (Figure 4).

Inflammatory cytokines TNF-α and IL-6 secreted by microglial M1 macrophages and Angptl2 have been shown to induce neuroinflammation [42,43] and participate in the progression of neurodegenerative diseases, such as Alzheimer’s disease [44,45].

Blue light has also been found to induce stress in the brain. Living organisms feel stress when exposed to blue light. Stress causes the secretion of stress hormones, i.e., glucocorticoids. Glucocorticoids reportedly inhibit new synapse formation and result in memory inhibition. Furthermore, in the relationship between brain microglia and stress, stress causes a rapid increase in brain epinephrine levels and enhances glucocorticoid levels in the brain [46,47]. In the brain, epinephrine acts through α- and β-adrenergic receptors to regulate learning and memory. Glucocorticoids easily enter the brain and regulate learning and memory via glucocorticoid receptors [48,49]. In the brain, microglia are crucial regulators of neurological function [50,51] and comprise several receptors for epinephrine and glucocorticoids [52]. Stress increases the number of microglia, impairing crosstalk with neurons and disrupting glutamate signaling [53]. Considering the immune response of microglia, stress induces a pro-inflammatory state in microglia, enhancing the expression of IL-1, IL-6, and TNF-α [54] and reducing levels of the anti-inflammatory cytokine IL-10 [55]. Accordingly, stress may cause memory impairment and related symptoms. Given that stress was not analyzed in the current study, it is necessary to also consider the blue light/stress axis.

**Figure 6:**
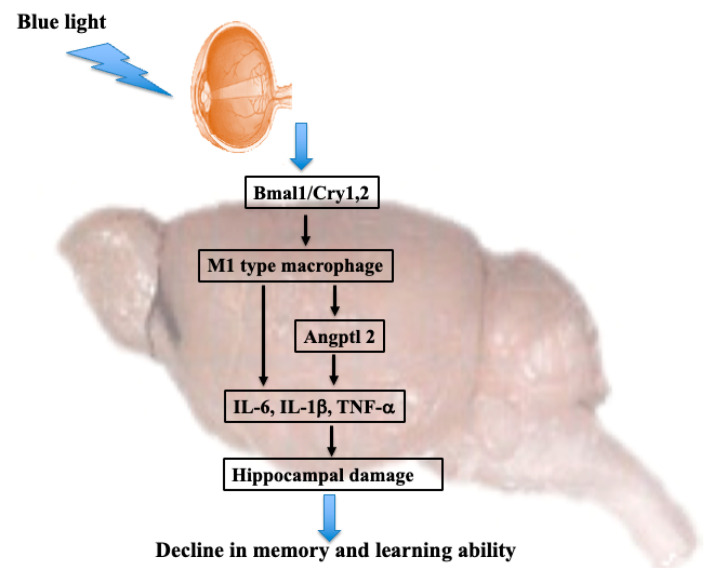
Mechanism of the effect of long-term blue light irradiation on memory and learning ability in mice.

Collectively, the results of the present study revealed that blue light regulates clock genes in the brain and induces microglial activity. Microglia shift to M1 macrophages and directly or indirectly induce the secretion of inflammatory cytokines, leading to a decline in learning and memory (Figure 6). These results indicate that staring directly at blue light from smartphones and computers for prolonged periods of time can damage the brain and suggest the need to protect the eyes from blue light.

We used mice in this study. Because mice are nocturnal animals, they are much more sensitive to blue light than humans. Additionally, mice have smaller eye dimensions compared to humans, which allows blue light to penetrate deeper. Conversely, humans are diurnal and less sensitive to blue light than mice. Furthermore, the circadian expression patterns of clock genes are also different between humans and mice. Therefore, in humans, the decline in learning ability due to long-term blue light irradiation of the eyes is not as pronounced as in mice. Furthermore, because mice are nocturnal, it has been thought that their visual acuity is not very well developed. However, it has been reported that visual perception is important for mice as well, and that they have many things in common with humans [56]. On the other hand, in addition to the fact that mice have poor visual acuity and little developed color vision, it is necessary to consider differences from humans, such as the animal’s morphological characteristics (position of the eyes on the face) and ecological environment. Therefore, there are limits to mouse research, but an important point in the future will be how we can bring it closer to humans.

## Data Availability

Data are available within the article.
